# Testicular differentiation in 46,XX DSD: an overview of genetic causes

**DOI:** 10.3389/fendo.2024.1385901

**Published:** 2024-04-24

**Authors:** Maria Tereza Martins Ferrari, Elinaelma Suelane do Nascimento Silva, Mirian Yumie Nishi, Rafael Loch Batista, Berenice Bilharinho Mendonca, Sorahia Domenice

**Affiliations:** ^1^ Disciplina de Endocrinologia e Metabologia, Laboratório de Hormônios e Genética Molecular/LIM42, Hospital das Clínicas da Faculdade de Medicina da Universidade de São Paulo, São Paulo, Brazil; ^2^ Laboratório de Sequenciamento em Larga Escala (SELA), Faculdade de Medicina da Universidade de São Paulo, São Paulo, Brazil

**Keywords:** differences of sex development (DSD), 46, XX testicular DSD, 46, XX ovotesticular DSD, gonadal development, ovary

## Abstract

In mammals, the development of male or female gonads from fetal bipotential gonads depends on intricate genetic networks. Changes in dosage or temporal expression of sex-determining genes can lead to differences of gonadal development. Two rare conditions are associated with disruptions in ovarian determination, including 46,XX testicular differences in sex development (DSD), in which the 46,XX gonads differentiate into testes, and 46,XX ovotesticular DSD, characterized by the coexistence of ovarian and testicular tissue in the same individual. Several mechanisms have been identified that may contribute to the development of testicular tissue in XX gonads. This includes translocation of *SRY* to the X chromosome or an autosome. In the absence of *SRY*, other genes associated with testis development may be overexpressed or there may be a reduction in the activity of pro-ovarian/antitesticular factors. However, it is important to note that a significant number of patients with these DSD conditions have not yet recognized a genetic diagnosis. This finding suggests that there are additional genetic pathways or epigenetic mechanisms that have yet to be identified. The text will provide an overview of the current understanding of the genetic factors contributing to 46,XX DSD, specifically focusing on testicular and ovotesticular DSD conditions. It will summarize the existing knowledge regarding the genetic causes of these differences. Furthermore, it will explore the potential involvement of other factors, such as epigenetic mechanisms, in developing these conditions.

## Introduction

Gonadal development is a fundamental step in forming the reproductive system, and several diseases are associated with atypical gonadal development. The determination and differentiation of gonads from the bipotential gonadal primordium can be triggered by a combination of genetic and environmental factors, making it a species-specific process among vertebrates (1).

In mammals, sex is determined by genetic heritage during fertilization. The differentiation of fetal bipotential gonads into testes or ovaries occurs through the action of specific genetic networks. These developmental pathways are typically distinct, mutually exclusive, and driven by a complex interchange of antagonistic genes (2). Changes in the dosage and/or spatiotemporal expression of sex-determining genes can lead to disruptions in the typical development of male or female gonads, causing differences in sex development (DSD). In rare conditions, testicular tissue can develop into an XX gonad, resulting in the condition called 46,XX ovotesticular or testicular DSD.

## Clinical presentation

### Testicular difference of sex development

Testicular DSD (T DSD) has an estimated frequency of 1:20,000 to 1:25,000 newborn boys. These conditions account for about 2% of cases of male infertility. In about 80% of affected individuals, the genital male phenotype appears typical at birth, but diagnosis usually occurs during or after puberty due to symptoms such as gynecomastia, hypogonadism, and infertility (3). However, in some cases, individuals may present with atypical external genitalia, which enables for earlier investigation and evaluation. The severity of the condition depends on the extent of testicular tissue development.

### Ovotesticular difference of sex development

Ovotesticular DSD (OT DSD) is a rare form of DSD, with an estimated incidence of 1:100,000 births (4). This condition is characterized by the presence of both male gonadal tissues, with well-developed seminiferous tubules, and female gonadal tissue, with primordial follicles, within the same individual. In some patients, both types of gonadal tissues may be present in the same gonad, which is referred to as an ovotestis (5). The 46,XX karyotype is the most commonly identified chromosomal pattern in OT DSD, accounting for 65 to 90% of patients (6–8).

Most of the affected individuals present with atypical genitalia at birth. Individuals assigned as males at birth might experience breast development and/or cyclic hematuria. Similarly, individuals assigned as females may exhibit breast development and menstrual irregularities and/or signs of masculinization (9–11).

Although most cases of TDSD and OTDSD are sporadic, there are reports in the literature of individuals with both conditions occurring in the same family. This suggests that a common genetic origin may contribute to the development of these conditions (12–15).

### Genetic regulation of gonad development

Gonad development initially follows a similar trajectory in both XX and XY fetuses, with a bipotential gonad being formed from the urogenital crest. After the formation of the bipotential gonad, the processes involved in sex determination guide the development of sex-specific gonadal structures. In male development, there is an interaction between a network of pro-testis genes that promote the differentiation of the bipotential XY gonads into testes. Conversely, in female development, a network of pro-ovarian genes interacts to differentiate the XX bipotential gonads into ovaries (16).

### Bipotential gonad

**Figure 1:**
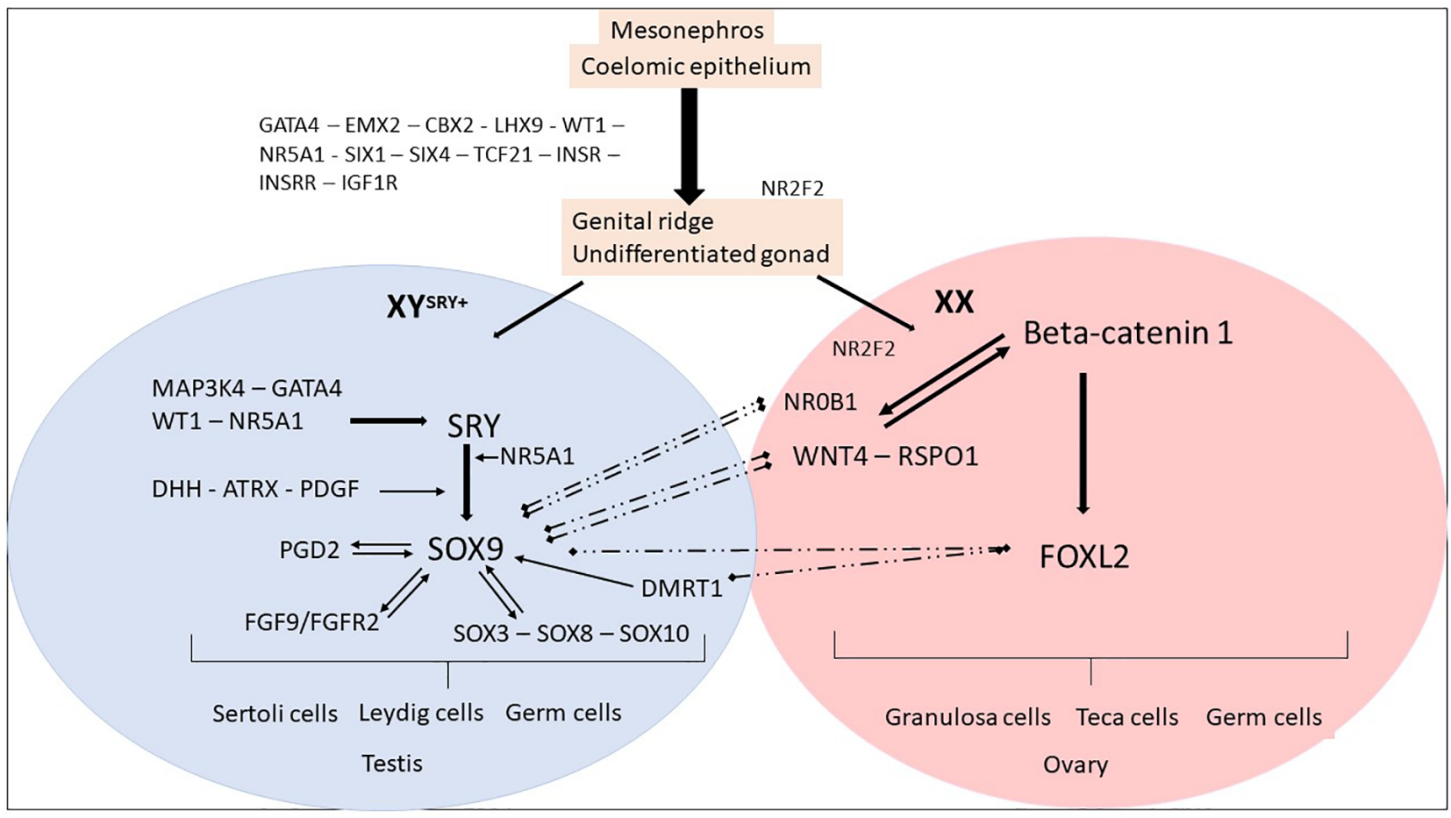
Genes and mechanisms of sex determination. The proliferation of cells from the mesonephros and coelomic epithelium leads to the formation of an undifferentiated and bipotential gonad. This process is regulated by several factors, including GATA4, EMX2, CBX2, LHX9, and WT1. These factors, in turn, regulate NR5A1, SIX1, SIX4, TCF21, and members of the IGF family, leading to the formation of an undifferentiated gonad. The process continues until the fate of the gonad is established, resulting in the formation of either an ovary or a testis. Ovarian differentiation. In the XX fetuses (absence of SRY), the expression of SOX9 remains low and other factors such as NR0B1, FOXL2, WNT4, and RSPO1 become dominant. The upregulation of WNT4 and RSPO1 leads to the activation of the canonical WNT signaling pathway, which in turn upregulates and stabilizes β-catenin. The activation of the WNT/β-catenin pathway plays a crucial role in the differentiation of the female gonad. NR2F2 has a role in maintaining a multipotent state in early supporting gonadal cells, which seems to be necessary for commitment to ovarian development. After birth, FOXL2 continues to suppress male-specific factors, including SOX9 and DMRT1. Testicular differentiation. In XY fetuses, the expression of SRY is triggered by MAP3K4, GATA4, WT1, and NR5A1. The presence of SRY and NR5A1 initiates the expression of SOX9, which leads to the differentiation of pre-Sertoli cells and subsequent Sertoli cells. Other members of the SOX family are also upregulated. SOX9 expression is maintained through positive feedback loops involving FGF9 and PGD2, as well as the regulation from WT1 and NR5A1. The increased expression of SOX9 prevails over NR0B1, FOXL2, WNT4, and RSPO1, promoting testicular differentiation. After birth, DMRT1 suppresses the female-specific factor FOXL2. These interactions between the male and female pathways remain essential throughout adulthood to maintain the gonadal identity.

In humans, the genital ridge first emerges between the fourth and fifth weeks of pregnancy. During this period, coelomic epithelial cells undergo proliferation on the ventromedial surface of the mesonephros. This proliferation process is tightly regulated by numerous genes and involves coordinated activity, which leads to the formation of bipotential gonads (17–19) (**Figure 1**).

In mice, null mutations in genes such *as Emx2* (Empty Spiracles Homeobox 2)*, Cbx2* (Chromobox protein homolog 2)*, Gata4* (GATA Binding Protein 4)*, Lhx9* (LIM homeobox 9)*, Wt1* (Wilms tumor 1)*, and Nr5a1* (Nuclear Receptor Subfamily 5 Group A Member 1) result in regression and changes in the development of the gonadal ridge. Coelomic epithelial cells differentiate into two distinct somatic precursor lineages: supportive cell precursors and steroidogenic cell precursors (20, 21).

Concurrently, primordial germ cells migrate from the yolk sac along the hindgut and dorsal mesentery to colonize the gonad (22). The interaction between somatic and germ cells and signaling from somatic cells is essential for the proliferation and differentiation of primordial germ cells. Furthermore, the female germ cells play a role in maintaining the ovary (23). Subsequently, the bipotential gonad differentiates into testis and ovary, respectively, through a sex-related genes antagonistic network.

### Genetic control of ovarian development

In bipotential gonadal tissue of XX individuals, the process of ovarian determination is initiated by a cooperative network of pro-ovarian genes, which includes *WNT4* (Wingless Type MMTV integration site family, member 4), *RSPO1* (R-Spondin1), and *FOXL2* (Forkhead box L2) (24–26) (**Table 1**). These factors not only activate genes required for ovarian development but also repress pro-testis gene expression (27). In XX individuals, *WNT4* and *RSPO1* initially direct ovarian determination by upregulating and stabilizing the beta-catenin signaling pathway. *CTNNB1* (Catenin Beta 1) essentially promotes germ cell proliferation and granulosa cell differentiation (25, 28). *RSPO1*, through *CTNNB1*, prevents *WNT4* degradation to maintain ovarian fate (25). FOXL2 expression is initiated in the supporting somatic cells of bipotential gonads, in conjunction with WNT4 and RSPO1 (**Figure 1**).

**Table 1:** Genes associated with testicular development in 46,XX DSD patients.

Gene		*Locus*	Protein	Protein action	46,XX DSD		
Symbol	Name				Phenotype	Condition	Proposed Mechanisms
***DMRT1***	Double sex, Mab3, Related transcription factor 1	9p24.3	DMRT1	Transcriptional factor	46,XX T DSD 46,XX OT DSD	Overexpression	Gene implicated in early gonadal development. In adult testis is required to maintain Sertoli cell identity
***FOXL2***	Forkhead transcriptional factor 2	3q23	FOXL2	Transcriptional factor	POI/BPES	Underexpression	Gene implicated in maintain granulosa cell transcriptional profiles. In adult ovaries is required to maintain granulosa cell identity
***FGF9***	Fibroblast Growth Factor 9	13q12.11	FGF9	Signaling molecule	46,XX male with hypospadias	Overexpression	Gene affecting later events. It is required for Leydig cell differentiation.
***NR0B1***	Nuclear receptor subfamily 0 group B member 1	Xp21.3	DAX1	Nuclear receptor transcription factor	46,XX T DSD 46,XX OT DSD	Underexpression	Gene affecting later events. It represses SF1 action.
***NR2F2***	Nuclear Receptor Subfamily 2 Group F Member 2	15q26.2	COUP-TFII	Nuclear receptor transcription factor	Syndromic 46,XX T DSD	Underexpression	Gene regulates cell fate during gonad development
***NR5A1***	Nuclear receptor subfamily 5 group A member 1	9q33	SF1	Nuclear receptor transcription factor	46,XX T DSD 46,XX OT DSD POI	Unknown	Gene implicated in early gonadal development in both sexes
***RSPO1***	R-spondin homolog 1	1p34.3	RSPO1	Signaling molecule	Syndromic 46,XX T DSD	Underexpression	Gene required for ovarian development
***SOX3***	SRY-related, HMG-box gene 3	Xq27.1	SOX3	Transcriptional factor	46,XX T DSD 46,XX OT DSD	Overexpression	Gene affecting later events – reinforces testis differentiation
***SOX9***	SRY-related, HMG-box gene 9	17q24.3	SOX9	Transcriptional factor	46,XX T DSD 46,XX OT DSD	Overexpression	Gene affecting later events – specification of Sertoli cell, promoting testicular differentiation
***SOX10***	SRY-related, HMG-box gene 10	22q13.1	SOX10	Transcriptional factor	46,XX T DSD 46,XX OT DSD	Overexpression	Gene affecting later events – reinforces testis differentiation
***SRY***	Sex-determining Region-Y chromosome	Yp11.3	SRY	Transcriptional factor	46,XX T DSD 46,XX OT DSD	Translocation	Gene affecting later events- required for testis development
***WNT4***	Wingless-type mmtv integration site family, member 4	1p35	WNT4	Member of the WNT signaling pathway	MRKH syndrome Serkal syndrome	Underexpression	Gene required for ovarian development
***WT1***	Wilms’ Tumor 1	11p13	WT1	Transcriptional factor	46,XX T DSD 46,XX OT DSD	Unknown	Gene implicated in early gonadal development in both sexes

T, testicular; OT, ovotesticular; POI, Premature ovarian insufficiency; GD, Gonadal dysgenesis, DDS, Dosage sensitive sex reversal, Adrenal hypoplasia; BPES, blepharophimosis-ptosis-epicanthus-inverse syndrome; MRKH, Mayer-Rokitansky-Kuster-Hauser syndrome; WAGR, Wilms tumor, aniridia, genitourinary anomalies, mental retardation syndrome).

*FOXL2* is required throughout ovarian development and into adulthood to maintain granulosa cell differentiation and support folliculogenesis (2, 29). Foxl2 performs these functions through several mechanisms, such as interacting with ovarian pathway genes*, Fst* (Follistatin) and *Cyp19a1* (cytochrome P450 family 19 subfamily A member 1) (30) and binding to a Sox9 enhancer to reduce Sox9 expression (31). *CTNNB1* also promotes the repression of *SOX9* expression. The genes involved in ovarian determination tend to show their expression a little later in the process of bipotential gonadal differentiation than the genes of the testicular pathway (32).

### Genetic control of testis development

In individuals with XY chromosomes, the *SRY* gene triggers the cascade of testicular differentiation (33), regulated by *WT1* (34), *NR5A1* (35), *CBX* (36), *GATA4* (37) and its co-factor *ZFPM2* (Zinc Finger Protein, FOG Family Member 2) (38), inducing the expression of the *SOX9* gene (39) (**Figure 1**). SOX9 expression is upregulated immediately after SRY expression in the supporting cells of the developing testis and marks their differentiation into Sertoli cells (40). Subsequently, *SOX9* plays a central role in regulating the expression of various genes involved in male sexual differentiation, such as *FGF9*/*FGF2R* (Fibroblast Growth Factor/Fibroblast Growth Factor Receptor 2) (41), *PTGDS* (Prostaglandin D2 Synthase) (42), and *AMH* (Anti-Mullerian Hormone). Like SRY, the activity of SOX9 is both necessary and sufficient to induce testis development in the genital ridges (43, 44) (**Figure 1**). Indeed, *SOX9* prevents the expression of genes inducing the ovarian differentiation, such as *RSPO1* and *FOXL2* (45, 46). Other genes, including *MAP3K1* (Mitogen-Activated Protein Kinase Kinase Kinase 1) (47, 48), *WWOX* (WW Domain Containing Oxidoreductase) (49), *DMRT1* (Doublesex and Mab-3 Related Transcription factor 1) (50) and *DHX37* (DEAH-Box Helicase 37) (51), have been added as participants in the testicular determination pathway after the identification of deleterious point mutations or copy number alterations associated with the phenotype of differences of testicular differentiation in humans and mice (45).

## Molecular mechanisms involved with the development of testicular tissue in the 46,XX gonads

### *SRY*-negative with insufficient expression of pro-ovarian genes

#### *WNT4* gene

**Figure 2:**
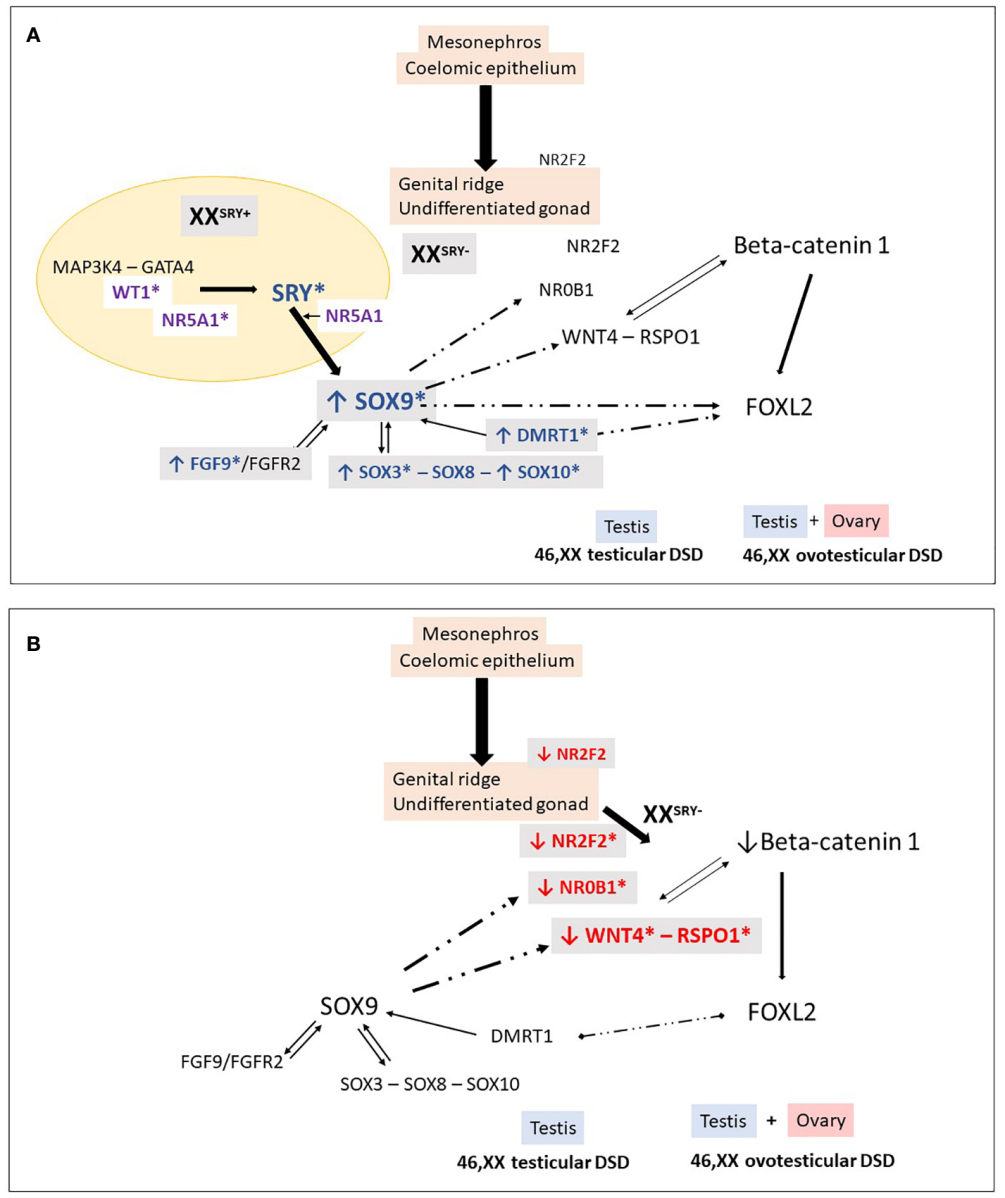
46,XX Testicular and ovotesticular DSD. Loss of the antagonistic balance of the RSPO1/WNT4/β-catenin pathway and the SRY/SOX9/FGF9 pathway can lead to the development of an abnormal gonad. **(A)** In XX individuals with Yp translocations and the presence of SRY, testicular differentiation can occur. In 46,XX SRY-negative individuals, testicular development may result from different conditions: overexpression of “pro-testicular” factors such as SOX9, SOX3, SOX10, FGF9, DMRT1, and **(B)** reduced expression of “pro-ovarian” factors such as RSPO1, WNT4, NR2F2. These changes in gene expression can be caused by an increase in the number of gene copies or their regulatory sequences. Additionally, in particular conditions, factors like WT1 and NR5A1 can also promote testicular development in 46,XX individuals. *Indicates genes associated with 46,XX testicular and ovotesticular DSD in humans.

*WNT4* (1p36.12) encodes a glycoprotein that plays multiple roles in ovarian differentiation and Müllerian duct formation (52) (**Table 1**). It is modulated by RSPO1 and acts by decreasing the phosphorylation and degradation of β-catenin. Increased levels of β-catenin antagonize SOX9, leading to upregulation of DAX1, which in turn antagonizes SF1 (53) (**Figure 2**). In mice with Wnt4 knockout, XX individuals exhibit virilization with the presence of Leydig-like cells in their gonad. While Wolffian ducts develop typically, Müllerian ducts are absent (54). In humans, heterozygous loss-of-function pathogenic variants in *WNT4* have been found in virilized 46,XX women, who presented excess ovarian androgens and atypical Mullerian duct development (55–58). Additionally, a homozygous *WNT4* pathogenic variant has been reported in a consanguineous family with a rare embryonic lethal syndrome known as SERKAL (SEx Reversion, Kidneys, Adrenal and Lung dysgenesis) syndrome (**Table 2**). This syndrome is characterized by *SRY*-negative 46,XX testicular or ovotesticular DSD, as well as adrenal hypoplasia, renal agenesis, and severe defects in the lungs and cardiovascular structures (68).

**Table 2:** *WNT4, RSPO1* and *NR2F2:* Genotype and clinical and gonadal characteristics of patients with *SRY*-negative 46,XX Testicular and Ovotesticular DSD reported in the literature.

Gene	Pathogenic mechanisms	Molecular findings	Diagnosis	External genitalia	Gonads	Reference
***WNT4***	Decreased Expression	c. 341C>T, (p.Ala114Val)	SERKAL syndrome	Atypical	Fe1: Dysgenetic testis Fe2: Ovotestis	Mandel H, 2008 (59)
***RSPO1***	Decreased Expression	c.108_109insG	46,XX Testicular DSD	Atypical, palmo-plantar keratosis	ND	Parma P, 2006 (60) Micali G, 2005 (61)
		Deletion of 2752bp (exon 4)	46,XX Testicular DSD	Atypical, palmo-plantar keratosis	ND	Parma P, 2006 (60) Vernole P, 2000 (62)
		Splice-donor site mutation (c.286 + 1G>A)	46,XX Ovotesticular DSD	Atypical, palmo-plantar keratosis	Ovotestis	Tomaselli S, 2008 (62)
		c.332G>A, (p.Cys111Tyr)	P1: 46,XX Testicular DSD P2: 46,XX Ovotesticular DSD	P1: Atypical, palmo-plantar keratosis P2: Atypical, palmo-plantar keratosis	P1: Dysgenetic testis P2: ND	Naasse Y, 2017 (63)
		c.43_43del A (p.Thr15Argfs*77)	46,XX Testicular DSD	Atypical, palmo-plantar keratosis	ND	Tallapaka K, 2018 (64)
***NR2F2***	Decreased Expression	c.103_109delGGCGCCC (p.Gly35Argfs*75)	P1: 46,XX DSD	P1: Male genitalia, non-palpable gonads	P1: ND	Bashamboo A, 2018 (65)
		c.97_103delCCGCCCG (p.Pro33Alafs*77)	P2: 46,XX DSD	P2: Atypical	P2: ND	
		c.97_103delCCGCCCG (p.Pro33Alafs*77)	P3: 46,XX Ovotesticular DSD	P3: Atypical	P3: Ovotestis (Bilateral)	
		3-Mb 15q26.2 (95127653_ 98146649)x1 deletion, arr[GRCh37]	46,XX Ovotesticular DSD	Atypical	Ovotestis	Carvalheira G, 2019 (66)
		c.23G>A, p.(Trp8*)	46,XX Testicular DSD	Atypical	Testis	Ganapathi M, 2023 (67)

SERKAL syndrome, SEx Reversion, Kidneys, Adrenal and Lung dysgenesis syndrome; ND, not described; P, Patient; Fe- Fetus.

#### *RSPO1* gene

*RSPO1* gene (1p34.3) encodes a secreted agonist protein of the canonical Wnt/β-catenin signaling pathway, that is widely expressed during fetal development (**Table 1**). RSPO1 plays a key role in gonad differentiation toward the ovary by synergizing the WNT4 to stabilize β-catenin in XX gonads (52, 59). In XX mice, the gonadal phenotype of the Rspo1 and the Wnt4 knockouts are strikingly similar: it ranges from small testes to ovotestes (26). RSPO1 is also expressed in fibroblasts and regulates the proliferation and differentiation of keratinocytes (69).

Homozygous deleterious *RSOP1* variants have been identified in *SRY*-negative 46,XX DSD patients with atypical genitalia and palmoplantar hyperkeratosis and increased susceptibility to squamous cell carcinoma of the skin (60, 62, 69, 70) (**Table 2**). These variants are typically located in the cysteine-rich furin domains of RSPO1, which are important for stabilizing cytosolic β-catenin. Dysregulation of β-catenin might result in the inhibition of Sox9 degradation and contribute to testis development (**Figure 2**).

Histological examination of gonads of two affected individuals reveals testicular structures with Leydig cell hyperplasia and ovotestes with small residual ovarian tissue, respectively (63, 70). The absence of RSPO1 also affects the skin microenvironment and epidermal integrity, contributing to an elevated risk of squamous cell carcinoma in palmoplantar regions exposed to frictional stresses (71). Some patients may also present with congenital microphthalmia, cataracts, coloboma of the iris and choroid, onychodystrophy, laryngeal carcinoma, and hearing impairment (60–62, 70, 72).

#### *NR2F2* gene

The *NR2F2* (Nuclear Receptor Subfamily 2 Group F Member 2) gene (15q26.2) encodes the chicken ovalbumin upstream promoter transcription factor 2 (COUP-TF2), which is an orphan nuclear receptor (**Table 1**). COUP-TFII plays important roles during embryogenesis, particularly in cell fate determination, organogenesis, angiogenesis, and metabolism (64, 73). It also plays a role in cell regeneration or dedifferentiation. High expression of COUP-TFII is observed in the mesenchymal component of various organs, including the heart, brain, kidney, adrenal cortex, genital tubercle, otocyst, periocular mesenchyme, optic stalk, and olfactory placode, during development and organogenesis (74). Knockout and heterozygous mice lacking COUP-TFII exhibit multiple vascular abnormalities, especially in the heart and brain. These abnormalities can lead to premature death, with embryonic mortality observed in COUP-TFII knockout mice and death occurring within the first few days of life in heterozygous mice (74).

In the gonadal ridges, COUP-TF2 acts as a “pro-ovary” and “anti-testis” factor (75). Previous studies suggest that the Nr2f2 repression is necessary for fetal Leydig cell differentiation (76).

Ferreira et al. demonstrated that the human NR2F2 is highly upregulated during bipotential gonad development, being detected in early somatic cells that precede the steroidogenic cell emergence in the undifferentiated gonad. The authors propose that COUP-TFII regulates cell fate during gonad development by modulating the WNT signaling pathway, Runx2 (RUNX family transcription factor 2) activity, and the expression of Pparg (Peroxisome Proliferator Activated Receptor Gamma) and Sox9. Impairment of its function might disrupt the transcriptional plasticity of early supporting gonadal cells. This disruption during early gonad development may cause early supporting gonadal cells to commit to the testicular pathway (77).

Less than 40 individuals with heterozygous pathogenic variants in *NR2F2* have been reported (78). Congenital heart defects are the most well-known phenotypes associated with its pathogenic variants, according to the expression pattern of COUP-TF2 (67, 73). However, the clinical features associated with *NR2F2* variants are variable. These features include intrauterine growth restriction (IUGR), congenital heart disease (CHD), congenital diaphragmatic hernia (CDH), blepharophimosis ptosis-epicanthus inversus syndrome (BPES), developmental delays, hypotonia, feeding difficulties, failure to thrive, congenital and acquired microcephaly, dysmorphic facial features (such as up-slanted or short palpebral fissures, micrognathia or retrognathia, low-set or dysplastic ears, hypertelorism, and full cheeks), renal failure, hearing loss, strabismus, asplenia, and vascular malformations. Genital anomalies and DSD have also been described (78).

The molecular mechanisms leading to testis development in some 46,XX patients with *COUP-TFII* loss-of-function have yet to be defined (77, 79). *NR2F2* pathogenic variants/deletion were found to be associated with five patients who had a syndromic form of *SRY*-negative 46,XX T/OT DSD (**Table 2**) (65, 66, 78, 79).

These patients presented with atypical genitalia (4/5), congenital diaphragmatic hernia (CDH) (3/5), blepharophimosis ptosis-epicanthus inversus syndrome (BPES) (3/5), and congenital heart disease (CHD) (2/5). Three of the patients had frameshift variants affecting the N-terminal region of the protein, specifically, p.Gly35Argfs*75 and p.Pro33Alafs*77 and the fourth patient had a *de novo* nonsense variant, p.Trp8* (78, 79).

(**Table 2**). In the fifth patient, a CGH array assay identified a 3-Mb 15q26.2 [arr(GRCh37) 95127653_98146649] x1 deletion that encompassed the entire *NR2F2* gene (65, 66).

Genotype-phenotype correlations cannot be identified, as individuals carrying identical *NR2F2* variants may present with variable phenotypic manifestations. In the case of 46,XX patients, a single-copy genomic deletion that encompasses the entire *NR2F2* gene may result in testicular tissue and atypical external genitalia in some cases, but in others, there may be no evidence of genital anomalies or DSD, despite the presence of other syndromic features (65, 66, 78, 80). These findings suggest that the phenotypic expression of NR2F2-related differences may be likely influenced by additional modifiers.

## Presence of *SRY* gene in the pro-ovarian genes pathway

*SRY* initiates the formation of male gonadal tissue from bipotential gonadal primordia by stimulating a cascade of related genes, the SRY-related HMG box-containing genes (SOX) (**Table 1**). These genes play an essential role in the differentiation of Sertoli cells and the development of the testes (81).

The main cause of 46,XX T DSD patients is related to a chromosomal rearrangement during paternal meiosis that leads to the translocation of the *SRY* from the paternal Y chromosome to the X chromosome or an autosome. In such cases, patients typically exhibit external and internal male genitalia (82).

In such cases, the genetic etiological diagnosis of 46,XX T DSD can be established using the fluorescence *in situ* hybridization (FISH) technique, which identifies a fluorescent signal indicating the sequence of the *SRY* translocated onto the X chromosome or autosome. Alternatively, the polymerase chain reaction (PCR) can be used to identify the presence of the *SRY* in the individual’s DNA being evaluated. Microarray analysis is also used to detect the presence of *SRY*.

It is worth noting that the formation of the testis can occur in 46,XX individuals, even in the absence of *SRY*, particularly among those who have dosage variations in HMG-box transcription factors (**Table 1**).

## *SRY*-negative with overexpression of pro-testicular genes

The increased expression of genes associated with male gonadal determination is a well-established etiological cause of 46,XX T/OT DSD patients. Among these genes, members of the SOX family play a significant role in this process.

### *SOX* family

The SOX (SRY-related HMG box) family of proteins is a group of transcriptional regulators that contain a highly conserved high-mobility group domain (83, 84). The high-mobility group domain was first identified in the *SRY* gene, and several genes from the *SOX* family have been linked to the etiology of differences of gonadal developmental in mammals.

#### *SOX9* gene

*SOX9* (17q24.3) is a transcription factor that plays a significant role in various tissues, including chondrocytes and testes (84) (**Table 1**). Studies investigating the relationship between phenotype and genotype in humans and mice have demonstrated that *SOX9* expression is a critical step in testis development, occurring downstream of *SRY*. SOX9 is responsible for the specification of Sertoli cells, which in turn initiates testicular differentiation and triggers the production of AMH (85, 86).

Overexpression of *SOX9*, often caused by gene duplications or copy number variations in the upstream promoter region, has been linked to testis determination in the absence of *SRY* (84, 87, 88) (**Figure 2**). In many cases of 46,XX T/OT DSD, *SOX9* duplications have been identified as the most commonly observed genetic cause, second only to *SRY* translocation (**Table 3**).

**Table 3:** *SOX9*: Genotype and clinical and gonadal characteristics of patients with *SRY*-negative 46,XX Testicular and Ovotesticular DSD reported in the literature.

Gene	Pathogenic mechanisms	Molecular findings	Diagnosis	External Genitalia	Gonads	Reference
***SOX9***	Increased expression	Duplication of *SOX9*	46,XX Testicular DSD	Atypical	ND	Huang B, 1999 (85)
			46,XX Testicular DSD	Male	Testis	Lee GM, 2014 (89)
		Duplication/Triplication of *SOX9* regulatory sequences	46,XX Testicular DSD	Atypical	Testis	Refai O, 2010 (90)
			46,XX Testicular DSD	Male	Testis	Cox JJ, 2011 (91)
			46,XX Testicular DSD	Male	Testis	Vetro A, 2011 (92)
			46,XX Ovotesticular DSD 46,XX Ovotesticular DSD 46,XX Ovotesticular DSD	P1: Atypical P2: Atypical P3: Atypical	P1: ND P2: Testis/ovary P3: Ovotestis/dysgenetic gonad	Benko S, 2011 (93)
			46,XX Testicular DSD	Hypospadias	ND	Xiao B, 2013 (94)
			46,XX Testicular DSD 46,XX Ovotesticular DSD 46,XX Testicular DSD	P1: Male P2: Atypical P3: Male	P1: ND P2: Ovotestis (bilateral) P3: ND	Vetro A, 2015 (95)
			46,XX Ovotesticular DSD	Atypical	Ovotestis/Testis	Kim GJ, 2015 (88)
			46,XX Testicular DSD 46,XX Testicular DSD 46,XX Testicular DSD	P1: Male P2: Male P3: Male	P1: Dysgenetic testis P2: Dysgenetic testis P3: ND	Hyon C, 2015 (96)
			46,XX Ovotesticular DSD	Male	Ovotestis	Ohnesorg T, 2017 (97)
			46,XX Ovotesticular DSD	Male	ND/ovotestis	Shankara N, 2017 (98)
			46,XX Ovotesticular DSD	Atypical	Ovary e ovotestis	López-Hernández B, 2018 (99)
			46,XX Testicular DSD 46,XX Ovotesticular DSD	P1: ND P2: ND	P1: Testis P2: Ovotestis	Croft B, 2018 (100)
			46,XX Ovotesticular DSD	Atypical	Testis/ovary	Mengen E, 2020 (101)
		Promoter-specific gain-of-function variant in the *SOX9*	46,XX Ovotesticular DSD	Atypical	Ovotestis/Ovary	Ushijima K, 2021 (102)

ND, not described; P, Patient; F, Family.

Many of these duplications involve a noncoding region spanning at least 24 kb, known as the RevSex region, located approximately 0.5-0.6 Mb upstream of the *SOX9* gene (89–100) (**Table 3**). This region is predicted to contain a human testis-specific enhancer, and the duplication of this enhancer drives the atypical expression of *SOX9*, leading to the activation of testicular differentiation (87, 92).

Ushijima et al. escribed an SRY-negative 46,XX OT DSD patient with a novel SOX9 missense variant (p.Glu50Lys) with promoter-specific gain-of-function (GoF) activity in *in vitro* studies. The authors demonstrated that E50K-SOX9 had (GoF) activity in the mTESCO-luciferase reporter, suggesting that it was due to change(s) in its bioactivity. GoF activity was observed in mTESCO-luc but not in mAmh-luc, thereby indicating that the acquisition of GoF activity was promoter-specific. To associate the promotor SOX9 variant with atypical expression of SOX9, and the beginning of testicular differentiation in the 46,XX OT DSD patient, mice carrying the Sox9 p.E50K were also generated and characterized. These mice, nevertheless, did not develop ovotestis (101). Such discordance of expressivity/phenotype among humans and mice are not limited to sox9/SOX9 (102) but are also described in other genes associated with DSD, including Nr5a1/NR5A1 (103) and Wt1/WT1 (104). The molecular mechanism of the promoter -specific GoF activity of E50K-SOX9 remains to be elucidated.

#### *SOX3* gene

*SOX3* (Xq27.1) is another member of the *SOX* gene family that is involved in gonadal development. It encodes a protein that is highly SRY-like, with an amino acid sequence similarity of 67% for the protein and 90% for the HMG DNA-binding domain (105) (**Table 1**). Studies in transgenic mice have shown that increased ectopic expression of Sox3 in undifferentiated gonads can lead to sex reversal in XX mice, with complete virilization of external genitalia observed in 77% of animals (106). These findings suggest that Sox3 hyperexpression acts as a counterpart of Sry, leading to increased expression of Sox9. Together with Nr5a1, Sox3 binds to the enhancer region of Sox9 (106).

Like the findings in mice, when increased expression of *SOX3* is observed in humans, this gene acts in conjunction with *NR5A1* to promote overexpression of *SOX9*. This phenomenon directs the gonads toward male determination (105) (**Figure 2**).

The duplication of the *SOX3* in a patient with *SRY*-negative 46,XX OT DSD was initially identified by Sutton et al. (105). Several other 46,XX patients with testicular development (T and OT) and duplications of the *SOX3* or in the regions located upstream of this gene have been reported (94, 107–114), supporting the importance of *SOX3* in testis development (**Table 3**).

A heterozygous deletion downstream of *SOX3* was also reported in an *SRY*-negative 46,XX infertility male. The authors of the study speculated that this deletion may play a role in the regulation of the *SOX3*, potentially resulting in increased expression of SOX3 (115).

#### *SOX10* gene

*SOX10* (22q13.1) is another gene closely correlated with *SOX9* in humans (**Table 1**). Initially expressed in neural crest cells during the embryonic period, it plays a critical role in their development. *SOX10* is also expressed in fetal gonads (116). In mice, the expression of Sox10 specifically in Sertoli cells strongly indicates its involvement in the testicular differentiation process and reinforces its role in the male pathway (117).

Studies in transgenic animal models have demonstrated that Sox10 overexpression causes sex reversal in XX mice (117). These studies demonstrated that the expression level of Sox10 is crucial in determining the gonadal phenotype. Complete testicular differentiation in all mice was observed in the lineage with higher levels of Sox10 expression, while the lineage expressing lower levels of the transgene showed only 30% of mice with complete sex reversal in the postnatal period. Interestingly, all fetuses from the second group (lower expression levels) were able to initiate Sertoli cell differentiation (presence of cells expressing Sox9 in XX transgenic gonads). In these gonads, cells committed to the female pathway, identified by the expression of Foxl2, were interspersed with Sox9-positive cells (117). This same pattern has been described in ovotestis in humans, as well as in mouse models of ovotestis development (118).

Similarly, gonadal, and reproductive system alterations have been reported in cases of partial duplication of chromosome 22q in 46,XX humans, a chromosomal region that contains *SOX10* (**Table 4**). Rare patients diagnosed with 46,XX T/OT DSD, both syndromic and non-syndromic, have also been described in the literature with chromosome 22 aneuploidies (116, 119–121) (**Table 4**).

**Table 4:** *SOX3* and *SOX10*: Genotype and clinical and gonadal characteristics of the patients with *SRY*-negative 46,XX Testicular and Ovotesticular DSD reported in the literature.

Gene	Pathogenic mechanisms	Molecular findings	Diagnosis	External Genitalia	Gonads	Reference
***SOX3***	Increased expression	Duplication of *SOX3*	46,XX Testicular DSD	P1: Male P2: Male P3: Male	P1: ND P2: ND P3: ND	Sutton E, 2011 (103)
			46,XX Testicular DSD	Atypical	ND	Moalem S, 2012 (105)
			46,XX Testicular DSD	Male	ND	Vetro A, 2015 (95)
			46,XX Ovotesticular DSD	Atypical	Ovotestis	Grinspon RP, 2016 (82)
			46,XX Testicular DSD	Hypospadias	ND	Tasic V, 2019 (108)
			46,XX Ovotesticular DSD	Atypical	Ovotestis	Zhuang J, 2021 (109)
			46,XX Ovotesticular DSD	Atypical	Ovotestis/Testis	Wei J, 2022 (110)
			46,XX Testicular DSD	Male and cryptorchidism	ND	Oroz M, 2022 (111)
			46,XX Ovotesticular DSD	P1: Atypical	P1: Ovotestis	Oliveira FM, 2023 (112)
			46,XX DSD	P2: Atypical	P2: Ovary	
		Rearrangement of *SOX3* regulatory sequences	46,XX Testicular DSD	Atypical	Testis	Mizuno K, 2014 (106)
			46,XX Testicular DSD	Male	Dysgenetic testis	Vetro A, 2015 (95)
			46,XX Ovotesticular DSD	Atypical	Testis/Ovary	Haines B, 2015 (107)
		Deletion located downstream of the *SOX3*	46,XX Testicular DSD	Male	ND	Qin S et al, 2022 (113)
***SOX10***	Increased expression	Duplication of *SOX10*	46,XX Ovotesticular DSD	Atypical	Testis/Ovary	Aleck KA, 1999 (119)
			46,XX Testicular DSD	Male	ND	Seeherunvong T, 2004 (114)
			46,XX DSD	Male	ND	Falah N, 2017 (117)
		Chromosome 22 - Triplication	46,XX Testicular DSD	Atypical	Dysgenetic testis	Nicholl RM, 1994 (118)

ND, not described; P, Patient; F, Family.

#### *DMRT1* gene

The *DMRT1* gene (9p24.3) encodes a transcription factor that plays a crucial role in sex determination and gonadal development in various species (**Table 1**). It possesses a zinc-finger-like DNA binding domain known as the DM (doublesex/MAB-3) domain. This domain allows DMRT1 to bind to specific DNA sequences and regulate the expression of genes involved in sex differentiation (122). DMRT1 expression has been observed in the undifferentiated human XY gonadal primordium. During the early fetal period (gestational weeks 8-20), it is primarily expressed in Sertoli cells, which play a crucial role in testicular development. In the second gestational trimester, childhood, and post-puberty, DMRT1 expression becomes more abundant in spermatogonia (123) This dynamic pattern of expression suggests that *DMRT1* plays a significant role in both the early and later stages of male gonadal development.

DMRT1 expression has indeed been detected in oogonia and oocytes during the early stages of ovarian development, up until gestational week 20. However, it is important to note that after the onset of meiotic germ cell division, DMRT1 expression becomes absent in these cells (123).

In contrast to *DMRT1* homologs in other vertebrates, mammalian DMRT1 seems to not be involved in the initial sex determination but is instead required for maintaining male gonadal fate (124, 125). Studies in mice have demonstrated that the loss of expression of certain key genes in postnatal life can lead to the reprogramming of Sertoli cells into granulosa cells and vice versa. This suggests that there is a level of plasticity in gonadal fate even after the typical formation of a testis or ovary (31, 124).

Ectopic expression of *DMRT1* has been shown to reprogram differentiated female granulosa cells into male Sertoli-like cells. DMRT1 functions in collaboration with other key male sex regulators like SOX9 to maintain and reprogram sexual cell fate. It acts as a singular transcription factor, by regulating gene expression and chromatin accessibility (126).

Bertini et al. reported a three-year-old boy who presented with a typical male phenotype and an *SRY*-negative 46,XX karyotype (127). The genetic study conducted showed a heterozygous *de novo* in tandem duplication of 50,221 bp on chromosome 9p. This duplication encompassed exons 2 and 3 of the *DMRT1* and was detected using MPLA, CGH-array analysis, and Sanger sequencing. The breakpoints of the duplication were in the intronic regions, and it did not disrupt the coding frame of DMRT1. To investigate other potential genetic factors contributing to the phenotype, a custom NGS panel and whole genome sequencing were performed, but no additional pathogenic or uncertain variants were found in genes known to be involved in pro-testis/anti-ovary gene cascades.

The identified duplication might have allowed *DMRT1* to escape the usual transcriptional repression that occurs in 46,XX fetal gonads, leading to the activation of the testicular determination cascade. Notably, no previous cases of *SRY*-negative 46,XX DSD associated with alterations in *DMRT1* have been reported thus far.

#### *FGF9* gene

The *FGF9* gene (13q12.1) is a signaling peptide involved in the development of various organs, including limbs, lungs, the adenohypophysis, and the gonadal ridges (**Table 1**). FGFs are typically considered paracrine factors and play important roles in tissue patterning and organogenesis during embryogenesis. The FGF9 subfamily, which signals from epithelium to mesenchyme, stimulates mesenchymal proliferation. In *Fgf9* knockout XY mice, gonadal development is severely impaired during embryonic and fetal life, leading to reproductive phenotypes ranging from different range of undervirilization to complete feminization of external genitalia (128).

In a study by Chiang et al., an *SRY*-negative 46,XX male with hypospadias and azoospermia was identified (129). Array-CGH analysis revealed duplicated regions on chromosomes 13q12.11 (21.143874–21.174184 Mb) and 13q31.1 (79.807500–79.813700 Mb). These duplicated regions encompassed the entire *FGF9* and *SPRY2* genes, respectively. The genomic gain of *FGF9* was hypothesized to result in FGF9 overexpression, which could explain testicular development instead of ovarian development. Additionally, *SPRY2* was previously related to a potential role in male sex organogenesis by controlling *FGF9* gene-induced mesonephric cell migration to the developing testis (130). The higher amount of FGF9 would interfere with the expression of WNT4 in the embryo, thereby impeding ovarian development in *SRY*-negative 46,XX males.

## *SRY*-negative with pathogenic mechanisms not completely comprehended

*WT1 gene* (11p13) is a transcription factor that encodes a zinc-finger protein (**Table 1**). It is widely expressed in the condensing mesenchyme, genital ridge, fetal gonads, renal vesicle, developing podocytes of the fetal kidney, and mesothelium (131). The *Wt1* and *Lhx9* (Lim homeobox 9) genes act as direct activators of the *Nr5a1* and play a critical role in the development of the undifferentiated gonad (132).

More than 30 protein isoforms originating from WT1 alternative splicing, alternative translation start sites, and different RNA editing are known. The alternative splice site in intron 9 allows WT1 isoforms with omission or inclusion of three amino acids [lysine-threonine-serine (KTS)] between the third and fourth zinc fingers. These isoforms regulate specific urogenital differentiation processes (133, 134).

Pathogenic *WT1* variants are associated with several phenotypes, including 46,XY and 46,XX DSD (135).

WT1 also plays a crucial role in the differentiation and maintenance of Sertoli cells, and this function is positively related to the testicular abnormalities observed in XY patients with pathogenic *WT1* variants (37).

The role of WT1 in ovarian development is not yet completely understood. In mice, Wt1 is essential for the maintenance of granulosa cells, and its inactivation leads to atypical ovary development, characterized by reduced ovary size and a fewer number of developing follicles (136, 137).

In *SRY*-negative 46,XX individuals with testicular and ovotesticular DSD, seven pathogenic variants of *WT1* have been identified (**Table 5**) (147–150). These variants affect the fourth zinc finger, which is a highly conserved region of the WT1 protein. Testicular development in this condition may be influenced by the inappropriate interaction between the mutated WT1 protein and the main ovarian determinant, beta-catenin 1. Additionally, studies have shown that pathogenic variants in exon 10 increase the expression of genes such as *SOX9, NR5A1, and DMRT1*, which are involved in the development of Sertoli cells. It has been suggested that these alterations could promote the sequestration of beta-catenin 1, leading to the upregulation of pro-testicular pathways (148, 149).

**Table 5:** *NR5A1*, *NR0B1* and *WT1*: Genotype and clinical and gonadal characteristics of the patients with *SRY*-negative 46,XX Testicular and Ovotesticular DSD reported in the literature.

Gene	Molecular findings	Diagnosis	External genitalia	Gonadal histology	Reference
***NR5A1***	c.274C>T, p.Arg92Trp	46,XX Testicular DSD	Atypical	Testes (Bilateral)	Domenice S, 2016 (138)
	c.274C>T, p.Arg92Trp	46,XX Ovotesticular DSD 46,XX Testicular DSD 46,XX Testicular DSD 46,XX Testicular DSD	F1(n=2): Atypical F2(n=1): Male, micropenis F3(n=1): Male, micropenis F4(n=1): Male, hypospadias	F1(n=2): Ovotestis (Bilateral) F2(n=1): ND F3(n=1): ND F4(n=1): Dysgenetic testis (Bilateral)	Bashamboo A, 2016 (139)
	c.274C>T, p.Arg92Trp	46,XX Ovotesticular DSD 46,XX DSD Testicular	P1: Atypical P2: Male	P1: Testis/ovotestis P2: Testis (Bilateral)	Igarashi M, 2016 (140)
	c.274C>T, p.Arg92Trp	46,XX Testicular DSD 46,XX Ovotesticular DSD 46,XX Testicular DSD	P1: Female, clitoromegaly P2: Atypical P3: Male	P1: Testis/streak P2: Ovotestis bilateral P3: Testis (Bilateral)	Baetens D, 2016 (141)
	c.275G>A, p.Arg92Gln	46,XX Ovotesticular DSD	Atypical	P1: Ovotestis (Bilateral)	Swartz JM, 2016 (142)
	c.274C>T, p.Arg92Trp	46,XX DSD	Atypical	ND	Takasawa K, 2017 (143)
	c.274C>T, p.Arg92Trp	46,XX Testicular DSD	P1: Male, non-palpable gonads	P1: ND	Knarston IM, 2019 (144)
	c.274C>T, p.Arg92Trp	46,XX Ovotesticular DSD	P2: Atypical	P2: Ovotestis (Bilateral)	
	c.274C>T, p.Arg92Trp	46,XX Testicular DSD	P3: Atypical	P3: ND	
	c.779C>T, p.Ala260Val	46,XX Ovotesticular DSD	P4: Atypical	P4: Ovotestis/ovary	
	c.274C>T, p.Arg92Trp	46,XX Testicular DSD	Atypical	Testes (Bilateral)	Askari M, 2020 (145)
***NR0B1***	80 kb microdeletion removing the regulatory and the *NR0B1* sequences	46,XX Ovotesticular DSD	Atypical	Ovotestis (Bilateral)	Dangle P, 2017 (146)
***WT1***	c.1453_1456del, p.Arg485Glyfs*14	46,XX Testicular DSD	Atypical	Testis (bilateral)	Gomes NL, 2019 (136)
	p. Arg495Gly	46,XX Testicular DSD	P1: Atypical	P1: Dysgenetic testis (bilateral)	Eozenou C, 2020 (137)
	p.Pro481Leufs*15	46,XX Testicular DSD	P2: Atypical	P2: Dysgenetic testis (bilateral)	
	p.Arg495Gln	46,XX Testicular DSD	P3: Atypical	P3: Dysgenetic testis (bilateral)	
	p.Arg495Gln	46,XX Ovotesticular DSD	P4: Atypical	P4: Ovotestis (bilateral)	
	p.Arg495Gln	46,XX Ovotesticular DSD	P5: Atypical	P5: Ovotestis (bilateral)	
	p.Ser478Thrfs*17	46,XX Ovotesticular DSD	P6: Atypical	P6: ND	
	p.Lys491Glu	46,XX Testicular DSD	P7: Male	P7: Testis (bilateral)	
	c.1437 A>G	46,XX DSD	Atypical	ND	Sirokha D, 2021 (147)
	p.Arg495Gln	46,XX Testicular DSD	Atypical	Testis (bilateral)	Kirino S, 2023 (148)

ND, not described; P, Patient; F, Family.

### *NR5A1* gene

*NR5A1* (9q33.3) encodes the steroidogenic factor 1 (SF-1), which is expressed in the developing urogenital ridge, hypothalamus, anterior pituitary gland, and steroidogenic tissues (**Table 1**). SF-1 plays a crucial role in controlling several steps of adrenal and gonadal development (138, 151). *NR5A1* variants are associated with a wide phenotypic spectrum of 46,XX, and 46,XY DSD (139, 140).

A single and recurrent variant in the *NR5A1* (c.C274T, p.Arg92Trp), present in a heterozygous state, was identified in several 46,XX OT/T DSD patients (**Table 5**) (140) (141–143, 145, 152). In the study by Askari et al. (152), the p.Arg92Trp variant was identified in a pair of siblings with 46,XX DSD (ovotesticular and testicular DSD patients), as well as in their father who had oligospermia. This further supports the notion that the *NR5A1* variant can play a role in the development of different gonadal phenotypes (145). Another variant was identified in the Arg92 codon, just by changing the amino acid to Glutamine (c.G275A, p.Arg92Gln) in a 46,XX OT DSD patient (153). The arginine 92 residue is in a highly conserved region of NR5A1, which is crucial for its interaction with DNA. A third variant (c.C779T, p.Ala260Val) in the *NR5A1* was identified in a single 46,XX OT DSD patient (144).

To date, 13 families consisting of 15 patients with 46,XX DSD, and deleterious *NR5A1* variants have been reported (**Table 5**). These patients exhibit a variable range of virilization in the external genitalia, including isolated clitoromegaly, hypospadias, male genitalia with micropenis and cryptorchidism, or male genitalia and cryptorchidism. Likewise, the gonadal tissues also exhibit a diverse range, from streak/dysgenetic gonads to ovotestis or testis, depending on the specific case.

The mechanism which these three variants activate the testicular development in 46,XX OT/T DSD carriers remain elusive. It is suggested that they reduce the inhibition of the expression of male pathway genes, such as *SOX9* and *AMH* (141, 143), by disrupting specific ovarian development signals, mainly in the WNT/β-catenin pathway (144, 153).

### *NR0B1* gene

*NR0B1* (Nuclear Receptor Subfamily 0 Group B Member 1) gene is located in the dosage-sensitive sex reversal (DSS) region at Xp21.2 (**Table 1**). It encodes an unusual orphan nuclear receptor that lacks the classic DNA-binding domain (154, 155). NR0B1/DAX1 is expressed in various tissues including the developing urogenital ridge, hypothalamus, anterior pituitary gland, adrenal glands, and gonads. It is known to have a role in both ovarian and testicular development, especially in spermatogenesis (156, 157) In mice, a coordinated expression of Nr0b1, Sry, and potentially other factors is necessary to upregulate Sox9 expression in precursor somatic cells. This coordinated expression is crucial for the development of Sertoli cells in the testes (158). These findings confirm an essential role for *NR0B1* in both Sertoli and Leydig cell function (157, 159). However, the phenotype of male mice lacking *Nr0b1* can vary depending on the strain due to the background-specific abundance of male-determining *Sry* gene transcripts. This means that the presence of different genetic backgrounds can lead to variability in the phenotypes of XY mice lacking Dax1 (Nr0b1) (160). Additionally, Nr0b1 can be upregulated by Wnt4 through the activation of the WNT/β-catenin pathway (161). Loss of function of NR0B1 causes X-linked primary adrenal insufficiency and hypogonadotropic hypogonadism (162, 163).

If normal levels of NR0B1 are crucial for testicular development and spermatogenesis, an excessive dosage of NR0B1 has been suggested to act as an anti-testicular factor (164) Xp21.1 duplications, which include *NR0B1* and testis-specific *MAGEB* genes, have been identified in some XY patients with gonadal dysgenesis. These duplications contribute to abnormalities in gonadal development and function (146, 155, 165–168).

Dangle et al. (169) identified a copy number rearrangement in an *SRY*-negative 46,XX OT DSD patient using microarray analysis (**Table 5**). This rearrangement involved an 80 kb microdeletion and disrupted the Xp21.2 DSS critical region. The condition not only resulted in the removal of the regulatory sequences and the *NR0B1* gene, but it also impacted the normal genomic organization. This disturbance led to modified gene expression patterns through a position effect (169).

## Epigenetics control of gonadal development

Studies have indeed shown that epigenetic profiles undergo dynamic changes during mammalian development, serving as a critical mechanism in determining cell fate decisions and facilitating cellular differentiation (170). Although knowledge about the involvement of epigenetic regulators in human gonadal development remains limited, their role is unquestionable (171, 172).

Regarding the expression of miRNAs in fetal gonads, it is widely recognized that they play a role in the regulation of proteins that are critically involved in gonad development (173, 174). Moreover, it is observed that several miRNAs exhibit a sexually dimorphic expression pattern in fetal gonads, indicating their potential involvement in directing cell fate decisions and maintaining cellular states (174).

In the ovary, the role of miRNAs in follicle assembly, growth, differentiation, and ovulation has been identified (175). Real et al. (176) described miR-124 as a promising candidate gene for mice ovarian development. They found that miR-124 potentially targets several genes involved in sex determination, including Sox9, in their 3’-UTR regions. The authors also demonstrated that inhibiting miR-124 in XX gonadal cells resulted in the ectopic expression of Sox9, suggesting that this miRNA may down-regulate Sox9 in female gonads during the critical period of sex determination. Furthermore, miR-124 exhibited differential up-regulation in XX mice gonads during early stages of differentiation, but not in XY mice gonads (176). In humans, no report of miRNA abnormalities was related to 46,XX DSD etiology.

Various studies have also presented evidence suggesting the involvement of methylation patterns in the process of gonadal determination (171). However, there is currently no direct confirmation of a link between abnormal methylation patterns and the etiology of 46,XX DSD. It is known that DNA methylation and histone modifications are actively involved in the spatiotemporal expression of Sry by making the enhancers and the promoter accessible for the binding of multiple transcription factors (16, 171, 177). Furthermore, methylation of the promoter/regulatory region directly impacts the expression of the Sox9 gene in the testis and ovary of mammals. The adult testis exhibits strong Sox9 expression, while site-specific methylation in the adult ovary could play a crucial role in reducing Sox9 gene expression (178).

Certainly, innovative studies will play a crucial role in establishing the involvement of epigenetic mechanisms in the etiology of 46,XX DSD. These studies will contribute to expanding our understanding of gonads determination.

## Conclusion

While our understanding of ovarian determination has significantly advanced, the process of testicular tissue development in an *SRY*-negative 46,XX gonad remains intriguing. It is worth noting that the majority of individuals with *SRY*-negative 46,XX testicular and ovotesticular DSD have not received a confirmed genetic diagnosis. This highlights the possibility of unknown genetic pathways or epigenetic mechanisms involved in these conditions. Further research and expansion of patient cohorts are needed to identify these other new members of the gonadal determination cascade.

## Author contributions

MF: Writing – review & editing. ES: Writing – original draft, Writing – review & editing. MN: Writing – original draft, Writing – review & editing. RB: Writing – original draft, Writing – review & editing. BM:Funding acquisition, Writing – original draft, Writing – review & editing. SD: Funding acquisition, Writing – original draft, Writing – review & editing.
